# Diagnosis and treatment of autoimmune encephalitis in Brazil: an urgent call to action

**DOI:** 10.1055/s-0044-1781442

**Published:** 2024-02-23

**Authors:** Lívia Almeida Dutra

**Affiliations:** 1 Faculdade Israelita de Ciências da Saúde Albert Einstein, Instituto do Cérebro do Hospital Israelita Albert Einstein, São Paulo SP, Brazil.

We read with interest the results from Morillos et. al, 1 describing the barriers to diagnosing and treating patients with autoimmune encephalitis (AIE) from a regional hospital in South Brazil. While their study focused on a small cohort of patients with encephalitis harboring high-risk and cell-surface antibodies, it sheds light on the complexities of managing AIE within Brazil's healthcare system, encompassing both public and private sectors.

The authors described that out of 17 patients meeting the criteria for possible AIE, only 11 received testing, which was subject to delays and additional restrictions imposed by the hospital's administrative board. Furthermore, the available test results were limited and did not align with current AIE testing guidelines. Notably, all patients in the cohort were under 50 years old, all three patients with anti-NMDAR encephalitis died, while most survivors experienced functional dependence and developed epilepsy.

These findings diverge from the prevailing depiction of AIE in the literature as a treatable condition with a favorable prognosis when promptly addressed. 2 Mortality in anti-NMDAR encephalitis is estimated to be around 10% 2 ; most AIE patients became seizure-free when adequately treated. 3 Indeed, results from Morillos et al. 1 reinforce the importance of treatment in the first four weeks of initial symptoms. 4 Adequate diagnosis is a cornerstone for the treatment and survival, as others have published.

AIE predominantly affects young individuals and is a costly condition. 5 Patients require extensive investigation, often ICU beds, and treatments such as plasmapheresis, IVIG, and rituximab. 6 The diagnosis and management of AIE pose significant challenges to low-income countries. A recent multicentric study conducted in Brazil reported a significant six-month delay in diagnosing patients with AIE. 7 Furthermore, Brazilian patients received fewer rituximab doses when compared with other patient cohorts. 7 These finding underscores a concerning trend of delayed access to appropriate treatment within the Brazilian healthcare system. Additionally, the observed underreporting of AIE cases from Latin America suggests a pervasive issue of underdiagnosis in the region, further complicating efforts to address this condition effectively. 8

Barriers to the diagnosis and treatment of AIE in Brazil stem not only from healthcare system underfunding and the absence of a national treatment protocol but also from the limited understanding of the disease by healthcare providers. AIE encompasses a spectrum of disorders characterized by antibodies targeting neuronal surface or glial antigens, with anti-NMDAR encephalitis being the most recognized subtype. 7 Presently, over 12 antibodies have been associated with AIE, i.e., anti-LGI1, anti-GAD, anti-GABA-AR, anti-GABA-BR, and others. Diagnosis requires tests using immunofluorescence in the rat brain, a technique named tissue-based assay (TBA), followed by confirmatory cell-based assay (CBA). Optimal diagnostic practices recommend screening serum and CSF for antibodies using the two techniques due to variations in sensitivity depending on the sample. 9 Unfortunately, despite specific clinical phenotypes and diagnostic clues, antibody positivity cannot be reliably predicted based solely on clinical presentation and ancillary investigations.

Commercial assays for AIE are available at exorbitant costs, rendering them inaccessible to many patients within the public healthcare system. In Brazil, testing for antineuronal antibodies is more expensive than performing a genetic panel or exome for any other disease. Some patients resort to out-of-pocket payments for these tests, exacerbating disparities in access to care. A significant issue in diagnosing AIE is that certain laboratories only perform CBAs for a limited number of antibodies, which may not adequately rule out the disease. Additionally, commercial assays can be limited in their accuracy; research has revealed that commercial CBAs may yield false negative results in 14% of cases and may produce false positive results in CSF samples. 10 11

Although AIE is considered a rare disease, with estimated prevalence of 7–13 cases per 100.000 inhabitants, 5 it is a frequent differential diagnosis of many neurological and psychiatric conditions such as first psychosis episode, catatonia, rapidly progressive dementia, developmental delay, refractory status epilepticus, infectious encephalitis, and abnormal movement disorders. 6 9 Given its treatable nature, many neurologists face the problem of inadequate diagnosis by treating empirically patients with steroids and IVIG, an approach that may add cost to the health care system.

The findings from Morillos et. al, 1 together with other studies by Brazilian researchers, highlight the urgent need to establish a standardized approach for diagnosing and treating AIE patients, incorporating cost-effectiveness analyses. Prioritizing early testing, timely treatment, and minimizing sequelae should be our goal in managing AIE within the Brazilian healthcare system.
